# Impact of diabetes on the predictive value of heart failure biomarkers

**DOI:** 10.1186/s12933-016-0470-x

**Published:** 2016-11-03

**Authors:** Nuria Alonso, Josep Lupón, Jaume Barallat, Marta de Antonio, Mar Domingo, Elisabet Zamora, Pedro Moliner, Amparo Galán, Javier Santesmases, Cruz Pastor, Dídac Mauricio, Antoni Bayes-Genis

**Affiliations:** 1 Heart Failure Clinic, Health Sciences Research Institute & University Hospital Germans Trias i Pujol, Badalona, Spain; 2 Department of Endocrinology and Nutrition, CIBER of Diabetes and Associated Metabolic Diseases (CIBERDEM), Health Sciences Research Institute and University Hospital Germans Trias i Pujol, Badalona, Spain; 3 Department of Medicine, Universitat Autonoma de Barcelona, Badalona, Spain; 4 Department of Biochemistry, Hospital Universitari Germans Trias i Pujol, Badalona, Spain; 5 Department of Biochemistry, Hospital Universitario Marqués de Valdecilla, Santander, Spain; 6 Department of Medicine, UAB, Hospital Universitari Germans Trias i Pujol, Carretera del Canyet s/n, 08916 Badalona, Spain; 7 Department of Medicine, UAB, Endocrinology and Nutrition Service, Hospital Universitari Germans Trias i Pujol, Carretera del Canyet s/n, 08916 Badalona, Spain

**Keywords:** Diabetes mellitus, Heart failure, Biomarkers, ST2, Prognostic

## Abstract

**Background:**

Patients with diabetes mellitus (DM) have an increased risk of developing heart failure (HF). Further, DM is associated with poor prognosis in patients with HF. Our aim was to determine whether DM has any impact on the predictive value of a multi-biomarker panel in patients with HF.

**Methods:**

We included 1069 consecutive ambulatory HF patients in the study: age 66.2 ± 12.8 years, 33.5 ± 13.3 left ventricular ejection fraction, 36% diabetic patients. We measured serum concentrations of N-terminal pro-brain natriuretic peptide (NT-proBNP), high-sensitivity troponin T (hs-TnT), ST2, galectin-3, high-sensitivity C reactive protein (hs-CRP), cystatin-C, soluble transferrin receptor (sTfR), and neprilysin and followed patients for 4.9 ± 2.8 years. Primary endpoints were all-cause and cardiovascular death.

**Results:**

During follow-up, 534 patients died; 283 died of cardiovascular causes. Diabetic subjects had higher mortality (57.7 vs. 45.6%, *p* < 0.001). NTproBNP (*p* = 0.07), hs-TnT (*p* < 0.001), galectin-3 (*p* < 0.001), and cystatin-C (*p* = 0.001) concentrations were higher in diabetic patients, whereas sTfR levels were lower (*p* = 0.005). There were no interactions between DM and NTproBNP, hs-TnT, galectin-3, hs-CRP, cystatin-C, sTfR, and neprilysin relative to risk prediction for all-cause or cardiovascular death. By contrast, ST2 significantly interacted with DM for all-cause (*p* = 0.02) and cardiovascular (*p* = 0.03) death. In diabetic patients, HRs for ST2 were 1.27 (95% CI 1.16–1.40, *p* < 0.001) and 1.23 (95% CI 1.09–1.39, *p* = 0.001) for all-cause and cardiovascular death, respectively. In nondiabetic patients, HRs for ST2 were 1.53 (95% CI 1.35–1.73, *p* < 0.001) and 1.64 (95% CI 1.31–2.05, *p* < 0.001) for all-cause and cardiovascular death, respectively. The multivariable Cox regression analysis showed that hs-TnT and ST2 were the only markers that were independently associated with both all-cause and cardiovascular mortality in patients with HF and diabetes. Moreover, in these patients, the combination of these two markers significantly increased discrimination as assessed by the area under the curve.

**Conclusions:**

Biomarkers used in the general population to predict the clinical course of heart failure are also useful in patients with diabetes. In these patients, among all the biomarkers analysed only hs-TnT and ST2 were independently associated with both all-cause and cardiovascular mortality.

## Background

Epidemiological studies have confirmed that the presence of diabetes mellitus (DM) is associated with an increased risk of developing heart failure (HF) [1, 2]. Indeed, HF is often the first cardiovascular manifestation observed in diabetic patients [3]. The increased risk of HF persists even after adjustment for confounders such as coronary artery disease and hypertension. Further, diabetic patients with HF have a poor prognosis [4].

It is challenging to stratify risk for patients with HF. Established risk factors such as left ventricular ejection fraction (LVEF), New York Heart Association (NYHA) functional class, comorbidities (DM, anemia, and renal insufficiency), and treatment strategies do not fully explain the mortality risk associated with HF patients [5–7]. Prognostication may be refined by measurement of biomarkers for different pathophysiological processes not reflected by established mortality risk factors [8] Circulating biomarkers measured in patients with HF are classified into seven pathophysiological pathways, including myocardial stretch, myocyte injury, extracellular matrix, inflammation, renal dysfunction, neurohormonal activation, and oxidative stress [9, 10]. In recent years, there is a growing interest in relation to the clinical usefulness of these biomarkers in patients with diabetes [11] as well as to their pathogenic role in the development of vasculopathy [12, 13]. Some of these biomarkers have been shown to be associated with the risk of diabetes [14–16] and their serum levels were described to be modified by medical treatment [17]. It is unclear whether systemic diseases such as DM may affect the predictive value of these HF biomarkers. Accordingly, our aim in the present study was to assess whether DM had any impact on the predictive value of a multi-biomarker panel in patients with HF. We included the following biomarkers: N-terminal pro-brain natriuretic peptide (NT-proBNP; myocardial stretch and neurohormonal activation), neprilysin (neurohormonal activation), galectin-3 (extracellular matrix), ST2 (inflammation, stretch, and extracellular matrix), high-sensitivity troponin T (hs-TnT; myocardial injury), cystatin-C (renal dysfunction), high-sensitivity C reactive protein (hs-CRP; inflammation), and soluble transferrin receptor (sTfR; oxidative stress).

## Methods

### Study population

The present study is a subanalysis of a previous investigation of the performance of different biomarkers in a well-established HF cohort [10–13]. From May 2006 to 2013, ambulatory patients treated at a multidisciplinary HF clinic were consecutively included in the study. Referral inclusion criteria and blood sample collection are described elsewhere [4–7]. All analyses of biomarkers were performed on the same blood sample, which had been stored at −80° without prior freeze–thaw cycles. All samples were obtained between 9:00 a.m. and 12:00 p.m. Serum concentrations of NT-proBNP (*N* = 1030), hs-TnT (*N* = 803), ST2 (*N* = 814), galectin-3 (*N* = 811), hs-CRP (*N* = 773 after we excluded 16 patients with levels ≥100 mg/dL), cystatin-C (*N* = 804), STfR (*N* = 794), and neprilysin (*N* = 1069) were measured.

All of the participants provided written informed consent, and the local ethics committee approved the study. All of the study procedures were in accordance with the ethical standards outlined in the Helsinki Declaration of 1975 (revised in 1983).

### Follow-up and outcomes

All patients made follow-up visits at regular, predefined intervals, and they made additional visits as required in cases of decompensation. The regular visitation schedule included a minimum of quarterly visits with nurses; biannual visits with physicians; and elective visits with geriatricians, psychiatrists, and rehabilitation physicians. Patients who did not attend the regular visits were contacted by telephone.

The primary outcomes of the present study were all-cause and cardiovascular death. A death was considered cardiovascular in origin if it was caused by HF (decompensated HF or treatment-resistant HF in the absence of another cause), sudden death (unexpected death, witnessed or not, of a previously stable patient with no evidence of worsening HF or any other cause of death), acute myocardial infarction (due to mechanical, haemodynamic, or arrhythmic complications), stroke (in association with recent acute neurological deficits), procedural (death after diagnostic or therapeutic procedures), and other cardiovascular causes (e.g., rupture of an aneurysm, peripheral ischaemia, or aortic dissection). Fatal events were identified from paper and electronic clinical records, from general practitioners, and by contacting the patients’ relatives. Data were verified by comparisons with the databases of the Catalan and Spanish health systems. Events were adjudicated by two of the authors (MD and JL). Follow-up was closed on 30 September 2015. Five patients were lost during follow-up and appropriately censored.

### DM diagnosis

A diagnosis of DM was made when one of the following criteria were met: (1) a diagnosis of DM was previously established and recorded in the patient’s electronic history, (2) fasting plasma glucose ≥126 mg/dL or HbA1c ≥6.5% identified by laboratory testing [18], or (3) the patient had a current prescription for oral hypoglycaemic medication or insulin. All the included patients in this study had type 2 diabetes.

### NTproBNP assay

NTproBNP levels were determined by an immunoelectrochemiluminescence method (Elecsys®; Roche Diagnostics, Switzerland). The assay had inter-run CVs of 0.9–5.5%.

### Hs-TnT assay

Hs-TnT levels were measured by electrochemiluminescence immunoassay on the Modular Analytics E 170 (Roche Diagnostics). The hs-TnT assay had an analytic range of 3–10,000 ng/L. At the 99th percentile value of 13 ng/L, the CV was 9%.

### ST2 assay

ST2 levels were measured from plasma samples with a high-sensitivity sandwich monoclonal immunoassay (Presage® ST2 assay; Critical Diagnostics, San Diego, CA, USA). The ST2 assay had a within-run coefficient of <2.5% and total CV of 4%.

### hsCRP assay

hsCRP concentrations were measured by particle-enhanced turbidimetry (CRPHS, ref. 04628918 190; Roche Diagnostics) on the automatic analyser Cobas 6000 (Roche Diagnostics). The linearity of the method was 0.15–20.0 mg/L, the detection limit was 0.15 mg/L, the functional sensitivity was 0.3 mg/L, and the interserial CV was <8.4%.

### Galectin-3 assay

For galectin-3 measurements, we used an enzyme-linked fluorescent assay (BioMerieux ref. 411191) on a mini-VIDAS® analyser (BioMerieux, France). The CV for the assay was <10%, the linearity was 3.3–100.0 ng/mL, and the limit of detection was 2.4 ng/mL.

### Cystatin-C assay

Cystatin C was measured by a nephelometric technique that assesses immune complex formation between cystatin and antiserum anticystatin-C attached to latex particles (Cystatin C Radim, ref. NPP42; Radim Group, Pomezia, Italy). The CV between assays was 2.9%.

### STfR assay

Soluble transferrin receptor concentrations were measured with a particle-enhanced turbidimetric immunoassay (Tina-quant Soluble Receptor Transferrin STFR, Roche Diagnostics) on the automatic analyser Cobas 6000 (Roche Diagnostics). The linearity of the method was 0.5–40.0 mg/L, the detection limit was 0.50 mg/L, and the interserial CV was <3.8%.

### Neprilysin assay

Human neprilysin (NEP) was measured with a modified sandwich immunoassay (HUMAN NEP/CD10 ELISA kit, ref. SK00724-01, lot no. 20111893; Aviscera Biosciences, Santa Clara, CA, USA). At a positive control value of 1.4 ng/mL, the intra- and interassay CVs were 3.7 and 8.9%, respectively.

### Statistical analysis

Categorical variables are expressed as percentages. Continuous variables are expressed as means (SD) or medians (quartiles Q1–Q3) for normal and non-normal distributions, respectively. Normal distribution was assessed with normal Q–Q plots. Differences between nondiabetic and diabetic patients were assessed by Chi-squared test, Student’s *t* test, and Mann–Whitney U test, as required. We also assessed differences in the levels of the different biomarkers between nondiabetic and diabetic patients (subsequent to log transformation) after adjustments for age, sex, body mass index, and estimated glomerular filtration rate.

We performed two Cox regression analyses, with all-cause or cardiovascular death as the dependent variable and the selected biomarker plus DM plus the interaction between the covariate biomarker and DM as independent covariables. In the Cox models, to fulfill the assumption of linearity of the covariables NT-proBNP, hs-TnT, ST2, hs-CRP, galectin-3, and sNEP, we used the logarithmic functions of NT-proBNP, hs-TnT, hs-CRP, galectin-3, and sNEP; the quadratic term of ST2; and log(hs-TnT). Afterwards, Cox regression analyses were performed separately for diabetic and nondiabetic patients for each biomarker. We used a 1SD increase, calculated jointly for the whole cohort, for HR calculations in the five logarithm-transformed variables. ST2 analyses were performed for every 10 ng/mL change. In patients with sNEP levels below the lower range of detection (0.250 ng/mL), a concentration of 0.249 ng/mL was introduced as a continuous variable. Survival curves for all-cause death were plotted for nondiabetic and diabetic patients based on the best cut-off points of ST2, obtained from AUC. As a sensitivity analysis of the 265 diabetic patients with all the biomarkers collected, we performed two comprehensive multivariable Cox regression analyses with all the biomarkers and also clinical variables, again for all-cause and cardiovascular death. Finally, a simple clinical model (age, sex, New York Association functional class, left ventricular ejection fraction and estimated glomerular filtration rate (CKD-EPI equation) was developed and discrimination evaluated (AUC) adding each one of the biomarkers and the best short combinations. Statistical analyses were performed with SPSS 15 (SPSS Inc., Chicago, IL, USA) and version 2.11.1 of the R statistical package (Foundation for Statistical Computing, Vienna, Austria). Two-sided *p* < 0.05 was considered significant.

## Results

We included 1069 consecutive ambulatory HF patients in the study. Table 1 lists the clinical characteristics of the total cohort and statistical differences based on the presence or absence of DM. The mean age was 66.2 ± 12.8 years, and 72% of the patients were men. The predominant HF etiology was ischaemic heart disease (51%), and the mean LVEF was 33.5 ± 13.3%. In total, 385 patients (36%) had DM. Upon inclusion, 198 and 151 patients were receiving oral antidiabetic medication and insulin, respectively; 39 patients were taking both. During follow-up, these numbers increased to 285 and 241, respectively (166 patients were receiving both treatments). As expected, many of the clinical characteristics were different between nondiabetic and diabetic patients (Table 1). For example, serum concentrations of five of the eight studied biomarkers were higher in diabetic patients: NTproBNP (*p* = 0.07), hs-TnT (*p* < 0.001), galectin-3 (*p* < 0.001), cystatin-C (*p* = 0.001), and sTfR (*p* = 0.005) (Table 1). After adjustment for age, sex, body mass index, and estimated glomerular filtration rate, NTproBNP (*p* = 0.01), hs-TnT (*p* < 0.001), and sTfR (*p* < 0.05) levels remained significantly higher in diabetic patients, while levels of galectin-3 (*p* = 0.1), cystatin-C (*p* = 0.86), hs-CRP (*p* = 0.62), sNEP (*p* = 0.61), and ST2 (*p* = 0.28) were no longer significantly different between the two groups.

**Table 1:** Clinical characteristics and treatment during follow-up categorised according to diabetic status

Characteristics	Nondiabetic *N* = 684	DM *N* = 385	*p* value
Age, years	65.3 ± 14	67.8 ± 10.3	0.002
Men	501 (73.2)	267 (69.4)	0.17
White	677 (99)	384 (99.7)	0.18
Aetiology			<0.001
Ischaemic heart failure	310 (45.3)	235 (61)	<0.001
Dilated cardiomyopathy	80 (11.7)	43 (11.2)	0.94
Hypertensive	60 (8.8)	37 (9.6)	0.50
Valvular	81 (11.8)	36 (9.4)	0.15
Alcohol	48 (7)	10 (2.6)	0.001
Other	105 (15.3)	24 (6.2)	0.01
HF duration, months	24 (3–70.8)	24 (4–71.6)	0.77
LVEF, %	33.7 ± 13.3	33.1 ± 13.2	0.61
NYHA class III–IV	154 (22.5)	107 (27.8)	0.54
Hypertension	392 (44.2)	286 (74.3)	<0.001
Peripheral arteriopathy	75 (11)	82 (21.3)	<0.001
COPD	125 (18.3)	56 (14.5)	0.12
BMI, Kg/m^2^	26.4 (23.9–29.7)	27.6 (25–31.2)	<0.001
Heart rate, bpm	71.4 ± 14.6	73 ± 13.5	0.07
Blood pressure, mmHg	125.8 ± 22.2	128.8 ± 23.4	0.04
eGFR, mL/min/1.73 m^2^	57.7 ± 27.2	50.9 ± 24.9	<0.001
Sodium, mmol/L	139.2 ± 3.4	137.9 ± 8.4	<0.001
Haemoglobin, g/dL	13.1 ± 1.9	12.5 ± 1.8	<0.001
Biomarkers			
NTproBNP, ng/L	1183 (494–2679)	1469 (629–3697)	0.007
hs-TnT, ng/L	19.1 (9.1–36.4)	28.6 (14.7–44.6)	<0.001
ST2, ng/mL	38 (30.3–49.9)	38.6 (31.3–52.6)	0.23
hs-CRP, mg/L	3.2 (1.3–8.3)	4.2 (1.4–8.7)	0.28
Galectin-3, ng/mL	15.6 (11.8–21.5)	17.4 (14–23.4)	<0.001
Cystatin-C, mg/L	1.28 (1.04.–1.73)	1.42 (1.13–1.89)	0.001
Neprilysin, ng/mL	0.63 (0.38–1.23)	0.66 (0.39–1.09)	0.71
STfR, mg/L	3.6 (2.8–4.6)	3.9 (3–4.9)	0.005
Treatments, *N* (%)			
ACEI or ARB	617 (90.2)	335 (87.0)	0.11
Beta-blocker	611 (89.3)	353 (91.7)	0.21
MRA	378 (55.3)	245 (63.6)	0.008
Loop diuretic	605 (88.5)	365 (94.8)	0.001
Digoxin	262 (38.3)	151 (39.2)	0.77
CRT	55 (8)	32 (8.3)	0.88
ICD	90 (13.2)	50 (13)	0.94

Data presented as mean ± SD, median (interquartile range), or *N* (%)

*ACEI* angiotensin converting enzyme inhibitor, *ARB* angiotensin II receptor blocker, *BMI* body mass index, *COPD* chronic obstructive pulmonary disease, *CRT* cardiac resynchronisation therapy, *DM* diabetes mellitus, *eGFR* estimated glomerular filtration rate (CKD-EPI equation), *HF* heart failure, *hs*-*CRP* high-sensitivity C reactive protein, *hs*-*TnT* high-sensitivity troponin T, *ICD* implantable cardiac defibrillator, *LVEF* left ventricular ejection fraction, MRA mineralcorticoid receptor antagonists, *NYHA* New York Heart Association, *NTproBNP* N-terminal pro-brain natriuretic peptide, *STfR* soluble transferrin receptor

During a mean follow-up period of 4.9 ± 2.8 years (6.6 ± 2.3 years for patients who did not die in the follow-up period), 534 patients died. Of the patients who died, 284 died from cardiovascular causes: 137 of progressive HF, 67 of sudden death, 26 of acute myocardial infarction, four of stroke, six during cardiovascular procedures, and 31 of other cardiovascular causes. One hundred and ninety-six patients died of noncardiovascular causes, while the cause of death was unknown in 54 patients. As expected, both all-cause and cardiovascular mortality were higher in diabetic patients (57.7 vs. 45.6%, *p* < 0.001 and 34.8 vs. 24.2%, respectively; *p* < 0.001).

**Fig. 1:**
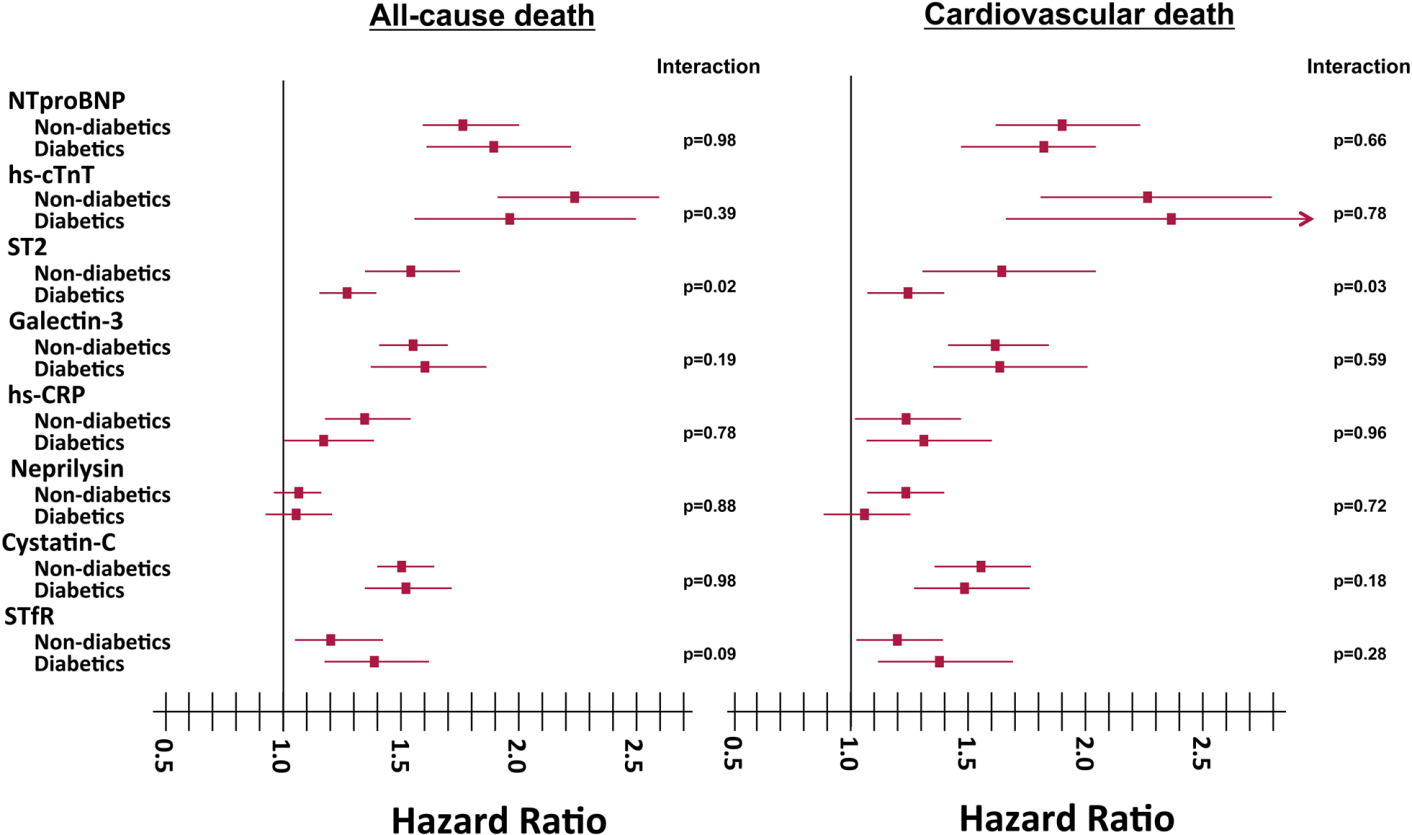
HRs and 95% CIs for biomarkers based on the presence or absence of DM, and statistical significance of the interaction covariate for DM and the biomarkers. A 1SD increase was used for HR calculations in the logarithm-transformed variables NTproBNP, hs-TnT, hs-CRP, cystatin-C, galectin-3, STfR, and neprilysin. ST2 analyses were performed for every 10 ng/mL change. Age was included as a covariate in the neprilysin analysis. For the quadratic form of ST2: *p* = 0.02 for all-cause death and *p* = 0.03 for cardiovascular death. For the quadratic form of log(hs-TnT): *p* = 0.82 for all-cause death and *p* = 0.1 for cardiovascular death. Interactions between DM and the quadratic form of ST2: *p* = 0.02 for all-cause death and *p* = 0.03 for cardiovascular death. Interactions between DM and the quadratic form of log(hs-TnT): *p* = 0.82 for all-cause death and *p* = 0.1 for cardiovascular death. hs-CRP, high-sensitivity C reactive protein; hs-TnT, high-sensitivity troponin T; NTproBNP, N-terminal pro-brain natriuretic peptide; STfR, soluble transferrin receptor

There were no interactions between the majority of the biomarkers and DM with respect to prediction of risk of all-cause or cardiovascular death (Fig. 1). However, ST2 significantly interacted with DM for all-cause (*p* = 0.02) and cardiovascular (*p* = 0.03) death.

**Fig. 2:**
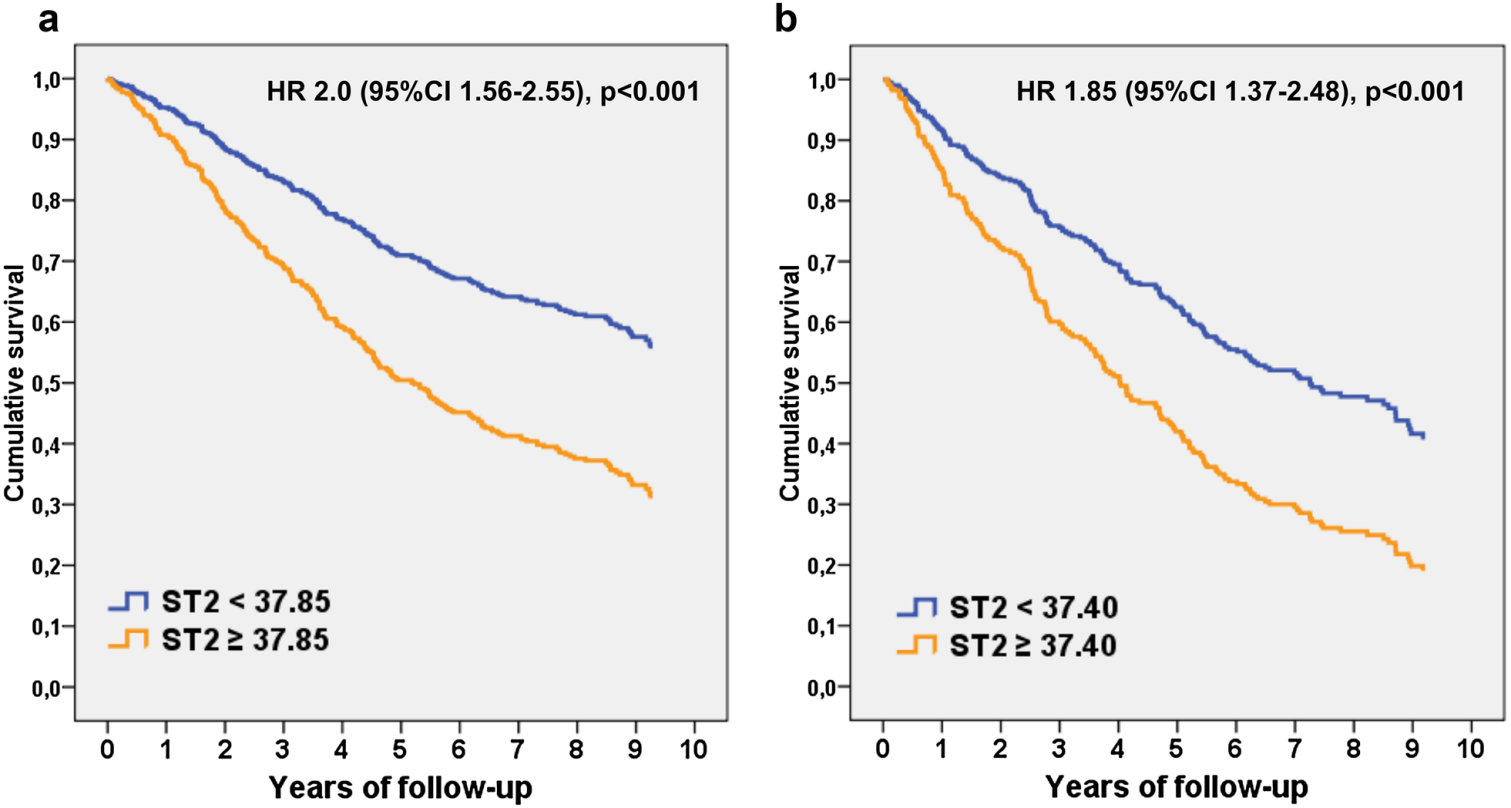
Cox regression survival curves for all-cause mortality relative to the best cut-off point of ST2. **a** Nondiabetic patients; **b** diabetic patients

Figure 1 shows the HRs and 95% CIs of Cox regression analyses for each biomarker for nondiabetic and diabetic patients, both for all-cause (left) and cardiovascular (right) death. In diabetic patients, the HRs for ST2 were 1.27 (95% CI 1.16–1.40, *p* < 0.001) and 1.23 (95% CI 1.09–1.39, *p* = 0.001) for all-cause and cardiovascular death, respectively. In nondiabetic patients, the HRs for ST2 were 1.53 (95% CI 1.35–1.73, *p* < 0.001) and 1.64 (95% CI 1.31–2.05, *p* < 0.001) for all-cause and cardiovascular death, respectively. Survival curves for diabetic and nondiabetic patients based on the best cut-off of ST2 values are shown in Fig. 2. Divergence of the curves and HRs are also mildly higher in nondiabetic than in diabetic patients. Table 2 shows, in a sensitivity analysis, multivariable Cox regression analyses performed in the 265 diabetic patients with all the biomarker studied. Hs-TnT, ST2 and cystatin-C remained statistically associated with all-cause death and hs-TnT, ST2 and galectin-3 with cardiovascular mortality. Finally, Discrimination analysis using a simple clinical model and the studied biomarkers and the best short combinations of them, according also to the previous multivariable analysis are shown in Table 3. Results show that the combination of two biomarker such as hs-TnT and ST2 significantly improved discrimination, as assessed by confidence intervals.

**Table 2:** Multivariable Cox regression analyses for risk of all-cause and cardiovascular death in diabetic patients

	All-cause death			Cardiovascular death		
	HR	95% CI	*p* value	HR	95% CI	*p* value
Age	1.04	1.02–1.06	<0.001	1.02	1.00–1.05	0.04
Female sex	0.77	0.54–1.11	0.16	0.70	0.43–1.13	0.15
Ischemic etiology of HF	0.90	0.64–1.27	0.56	0.98	0.63–1.52	0.92
LVEF	1.01	1.00–1.03	0.02	1.02	1.00–1.03	0.03
NYHA functional class	1.83	1.37–2.44	<0.001	1.75	1.18–2.58	0.005
eGFR	1.00	0.99–1.02	0.55	1.00	0.99–1.01	0.69
Hemoglobin	1.02	0.96–1.13	0.72	1.01	0.89–1.15	0.85
ACEI or ARB treatment	0.78	0.47–1.28	0.32	0.76	0.39–1.49	0.42
β-blocker treatment	0.93	0.52–1.65	0.79	1.06	0.49–2.30	0.89
NT-proBNP^a^	1.15	0.93–1.43	0.21	1.24	0.93–1.65	0.15
Hs-TnT^a^	1.32	1.08–1.63	0.008	1.53	1.13–2.05	0.005
ST2^b^	1.20	1.09–1.33	<0.001	1.18	1.03–1.34	0.01
Neprilysin^a^	0.99	0.85–1.16	0.91	0.99	0.80–1.22	0.94
Galectin-3^a^	1.12	0.92–1.37	0.25	1.27	1.03–1.57	0.02
Cystatin-C^a^	1.31	1.11–1.54	0.001	1.22	0.82–1.82	0.32
Hs-CRP^a^	0.91	0.78–1.06	0.24	1.05	0.85–1.29	0.65
STfR^a^	1.06	0.88–1.28	0.54	1.06	0.83–1.34	0.65

All *p* values are those found in the last step remaining in the model. *p* value for (ST2)^2^: All-cause mortality, p = 0.005; Cardiovascular mortality, p = 0.08. *p* value for (log-hs-TnT)^2^: All-cause mortality, p = 0.02; Cardiovascular mortality, p = 0.007

*ACEI* angiotensin converting enzyme inhibitor, *ARB* angiotensin II receptor blocker, *eGFR* estimated glomerular filtration rate (CKD-EPI equation), *hs*-*CRP* high-sensitivity C reactive protein, *hs*-*TnT* high-sensitivity troponin T, *LVEF* left ventricular ejection fraction, *NYHA* New York Heart Association, *NTproBNP* N-terminal pro-brain natriuretic peptide, *STfR* soluble transferrin receptor

^a^Log-transformed and per 1 SD

^b^Per 10 ng/mL

**Table 3:** C-statistics for risk of all-cause and cardiovascular death in diabetic patients

	All-cause death		Cardiovascular death	
	AUC	95% CI	AUC	95% CI
Clinical model	0.733	0.683–0.783	0.644	0.589–0.707
+NT-proBNP	0.751	0.702–0.800	0.666	0.606–0.624
+Hs-TnT	0.790	0.735–0.844	0.671	0.606–0.637
+ST2	0.790	0.735–0.846	0.646	0.579–0.713
+Neprilysin	0.733	0.683–0.783	0.648	0.589–0.707
+Galectin-3	0.780	0.724–0.835	0.650	0.583–0.716
+Cystatin-C	0.781	0.725–0.836	0.647	0.580–0.715
+Hs-CRP	0.769	0.710–0.827	0.639	0.571–0.707
+STfR	0.766	0.707–0.824	0.633	0.565–0.701
+Hs − TnT + ST2	0.811	0.759–0.863	0.683	0.617–0.748
+Hs − TnT + ST2 + NT-proBNP	0.810	0.757–0.863	0.690	0.625–0.754
+Hs − TnT + ST2 + cystatin-C	0.812	0.759–0.864	–	–
+Hs − TnT + ST2 + galectin-3	–	–	0.699	0.635–0.763

Clinical model: age, sex, New York Heart Association functional class, left ventricular ejection fraction, and estimated glomerular filtration rate (CKD-EPI equation); *hs*-*CRP* high-sensitivity C reactive protein, *hs*-*TnT* high-sensitivity troponin T, *NTproBNP* N-terminal pro-brain natriuretic peptide, *STfR* soluble transferrin receptor

## Discussion

The present study was performed in a large cohort of ambulatory patients with HF and included a long-term follow-up. The three main findings were as follows. First, the levels of most of the biomarkers that we studied were higher in diabetic patients; however, when we adjusted for confounders, only NT-proBNP, hs-TnT, and sTfR were significantly higher in diabetic patients. Secondly, independent of the finding stated above, we did not find any differences between nondiabetic and diabetic patients for most of the biomarkers with respect to prognostic value for all-cause and cardiovascular mortality. Finally sST2 which was a significantly predictor for both all-cause and cardiovascular death in both diabetic and nondiabetic subjects was associated with an appreciably higher risk in the nondiabetic patients. This biomarker together with hs-TnT were the only markers that were independently associated with both all-cause and cardiovascular mortality in the diabetic patients of our cohort. Some of the biomarkers that we investigated have been shown in previous studies to be predictors of the incidence of cardiovascular events and prognosis in diabetic patients and some of them have also shown to help in distinguishing those individuals with a high risk of CV disease among diabetic subjects [19]. In elderly patients with DM, NT-proBNP is a strong independent predictor of short-term cardiovascular mortality, including patients without preexisting cardiovascular disease [20]. Also, in type 2 diabetic patients with microalbuminuria but without known coronary artery disease, NT-proBNP has been shown to be strongly associated with future CV events [17]. Moreover, hs-TnT levels in diabetic subjects were recently described as the strongest predictor (in addition to sST2 and hs-CRP) of short-term outcome in patients with stable coronary artery disease [21]. Also in type 2 diabetic patients, normal hs-TnT level has a high negative predictive value for future adverse CV events [22]. In the present study there were no interactions between the majority of the biomarkers and DM with respect to prediction of risk of all-cause or cardiovascular death. However, ST2 significantly interacted with DM for all-cause and cardiovascular death being associated with a higher risk in nondiabetic compared to diabetic patients. Experimental and human studies have suggested relationships between ST2 and myocardial stretch, fibrosis, adverse cardiac remodelling, inflammation, impaired haemodynamics, and vascular disease [23]. Indeed, in experimental studies ST2 appears to play a pivotal role in LV remodeling; this process comprises changes in cardiac structure, myocardial composition, myocyte deformation, and multiple biochemical and molecular alterations that impact heart function and reserve capacity [24]. The currently available data are insufficient to allow us to determine conclusively whether the value of ST2 as a marker of cardiac remodelling is different in patients with and without diabetes. That is, patients with diabetes may be affected by other variables such as advanced glycation end products, inflammatory markers, or microvascular damage that may be involved in the remodelling process and thus could explain the difference in the prognostic value of ST2 concentrations between patients with and without DM. On the other hand, the source of ST2 is not well established; although it is known to be produced by both cardiac fibroblasts and cardiomyocytes in response to injury or stress, nonmyocardial production by endothelial cells has also been suggested [25]. The multivariable Cox regression analysis showed that ST2 together with hs-TnT were the only biomarkers analysed in the present study that were independently associated with heart failure prognosis (all-cause and cardiovascular mortality) in diabetic patients. Moreover, the combination of two biomarkers (ST2 together with hs-TnT) increased the predictive value of HF prognosis compared to what is obtained when only one of them is determined.

Diabetes is widely recognized as a significant risk factor for the development of HF and is an independent risk factor for increased mortality among individuals with HF. Indeed, HF is one of the most common initial manifestation of cardiovascular disease in patients with T2D [3]. Thus, it is of clinical interest to investigate whether the biomarkers used for assessing the prognosis of heart failure perform similarly in diabetic and non-diabetic subjects (both with regard to its serum concentrations and as to its prognostic value). To our knowledge there are no studies to date that have specifically addressed whether current predictive biomarkers used in the general population with heart failure are also useful in patients with diabetes. Our conclusion is that, in our cohort of HF patients, biomarkers used in the general population to predict the clinical course of heart failure are also useful in patients with diabetes. In these patients, among all the biomarkers analysed only hs-TnT and ST2 were independently associated with both all-cause and cardiovascular mortality. The use of a combination of biomarkers (multimarker strategy) in predicting the risk of death seems to be in diabetic patients as good as in the general population with HF [10], improving discrimination, mainly when two biomarkers are combined.

### Limitations

A limitation of this study is that most of the diagnoses of DM were made from clinical records, treatment regimens, and, for some patients, blood chemistry data alone. However, most previous diabetes studies used this form of data acquisition. Indeed, patients were classified into nondiabetic and diabetic subgroups according to their baseline diagnosis and no data on new-onset diagnosis of DM during follow-up were considered. Additionally, we could not assess the impact of diabetes duration as a powerful contributing factor in these patients. Although our study participants were drawn from the general population attending an HF unit, the unit is located at a tertiary university hospital and the cohort patients were mainly male and of ischaemic aetiology. The great majority had been admitted to a hospital in the previous year or had depressed LVEF. Therefore, we cannot disregard the possibility of bias due to selection of patients who may not necessarily represent the general HF population.

## Conclusions

DM has little impact on the predictive power of the HF biomarkers NT-proBNP, hs-TnT, galectin-3, hs-CRP, cystatin-C, sTfR, and neprilysin. Only ST2, which had predictive value in both diabetic and nondiabetic patients, was associated with a higher risk in nondiabetic patients.

## Abbreviations

DM: diabetes mellitus
HF: heart failure
hs-TnT: high-sensitivity troponin T
hs-CRP: high-sensitivity C reactive protein
sTfR: soluble transferrin receptor
LVEF: left ventricular ejection fraction
NYHA: New York Heart Association
NT-proBNP: N-terminal pro-brain natriuretic peptide

## References

[1] Kannel WB, McGee DL (1979). Diabetes and cardiovascular disease. The Framingham study. JAMA.

[2] Nichols GA, Hillier TA, Erbey JR, Brown JB (2001). Congestive heart failure in type 2 diabetes: prevalence, incidence, and risk factors. Diabetes Care.

[3] Shah AD, Langenberg C, Rapsomaniki E, Denaxas S, Pujades-Rodriguez M, Gale CP (2015). Type 2 diabetes and incidence of cardiovascular diseases: a cohort study in 1.9 million people. Lancet Diabetes Endocrinol.

[4] Bertoni AG, Hundley WG, Massing MW, Bonds DE, Burke GL, Goff DC (2004). Heart failure prevalence, incidence, and mortality in the elderly with diabetes. Diabetes Care.

[5] Bouvy ML, Heerdink ER, Leufkens HG, Hoes AW (2003). Predicting mortality in patients with heart failure: a pragmatic approach. Heart.

[6] Levy WC, Mozaffarian D, Linker DT, Sutradhar SC, Anker SD, Cropp AB (2006). The seattle heart failure model: prediction of survival in heart failure. Circulation.

[7] Pocock SJ, Wang D, Pfeffer MA, Yusuf S, McMurray JJ, Swedberg KB (2006). Predictors of mortality and morbidity in patients with chronic heart failure. Eur Heart J.

[8] Gravning J, Askevold ET, Nymo SH, Ueland T, Wikstrand J, McMurray JJ (2014). Prognostic effect of high-sensitive troponin T assessment in elderly patients with chronic heart failure: results from the CORONA trial. Circ Heart Fail.

[9] Braunwald E (2008). Biomarkers in heart failure. N Engl J Med.

[10] Lupón J, de Antonio M, Vila J, Peñafiel J, Galán A, Zamora E (2014). Development of a novel heart failure risk tool: the Barcelona bio-heart failure risk calculator (BCN bio-HF calculator). PLoS ONE.

[11] Kranendonk ME, de Kleijn DP, Kalkhoven E, Kanhai DA, Uiterwaal CS, van derGraaf Y, Pasterkamp G (2014). Extracellular vesicle markers in relation to obesity and metabolic complications in patients with manifest cardiovascular disease. Cardiovasc Diabetol.

[12] Gupta DK, Wang TJ (2015). Natriuretic peptides and cardiometabolic health. Circ J.

[13] Darrow AL, Shohet RV (2015). Galectin-3 deficiency exacerbates hyperglycemia and the endothelial response to diabetes. Cardiovasc Diabetol.

[14] Parrinello CM, Lutsey PL, Ballantyne CM, Folsom AR, Pankow JS, Selvin E (2015). Six-year change in high-sensitivity C-reactive protein and risk of diabetes, cardiovascular disease, and mortality. Am Heart J.

[15] Lin YH, Zhang RC, Hou LB, Wang KJ, Ye ZN, Huang T, Zhang J, Chen X, Kang JS (2016). Distribution and clinical association of plasma soluble ST2 during the development of type 2 diabetes. Diabetes Res Clin Pract.

[16] Coglianese EE, Larson MG, Vasan RS, Ho JE, Ghorbani A, McCabe EL, Cheng S, Fradley MG, Kretschman D, Gao W, O’Connor G, Wang TJ, Januzzi JL (2012). Distributionand clinical correlates of the interleukin receptor family member soluble ST2 in the Framingham heart study. Clin Chem.

[17] von Scholten BJ, Reinhard H, Hansen TW, Lindhardt M, Petersen CL, Wiinberg N, Hansen PR, Parving HH, Jacobsen PK, Rossing P (2015). Additive prognostic value of plasma N-terminal pro-brain natriuretic peptide and coronary artery calcification for cardiovascular events and mortality in asymptomatic patients with type 2 diabetes. Cardiovasc Diabetol.

[18] American Diabetes Association (2015). Classification and diagnosis of diabetes. Diabetes Care..

[19] Gori M, Gupta DK, Claggett B, Selvin E, Folsom AR, Matsushita K (2016). Natriuretic peptide and high-sensitivity troponin for cardiovascular risk prediction in diabetes: the atherosclerosis risk in communities (ARIC) study. Diabetes Care.

[20] Bruno G, Landi A, Barutta F, Ghezzo G, Baldin C, Spadafora L (2013). N-terminal probrain natriuretic peptide is a stronger predictor of cardiovascular mortality than C-reactive protein and albumin excretion rate in elderly patients with type 2 diabetes: the Casale Monferrato population-based study. Diabetes Care.

[21] Lepojärvi ES, Piira OP, Kiviniemi AM, Miettinen JA, Kenttä T, Ukkola O (2016). Usefulness of highly sensitive troponin as a predictor of short-term outcome in patients with diabetes mellitus and stable coronary artery disease (from the ARTEMIS study). Am J Cardiol.

[22] Yiu KH, Lau KK, Zhao CT, Chan YH, Chen Y, Zhen Z, Wong A (2014). Predictive value of high-sensitivity troponin-I for future adverse cardiovascular outcome in stable patients with type 2 diabetes mellitus. Cardiovasc Diabetol.

[23] Pascual-Figal DA, Lax A, Perez-Martinez MT, del Carmen Asensio-Lopez  M, Sanchez-Mas J (2016). GREAT Network. Clinical relevance of sST2 in cardiac diseases. Clin Chem Lab Med.

[24] Rodrigues PG, Leite-Moreira AF, Falcão-Pires I (2016). Myocardial reverse remodeling how far can we rewind?. Am J Physiol Heart Circ Physiol.

[25] Bartunek J, Delrue L, Van Durme F, Muller O, Casselman F, De Wiest B, Croes R, Verstreken S, Goethals M, de Raedt H, Sarma J, Joseph L, Vanderheyden M, Weinberg EO (2008). Nonmyocardial production of ST2 protein in human hypertrophy and failure is related to diastolic load. J Am Coll Cardiol.

